# Prevalence and associated risk factors for noma in Nigerian children: a systematic review and meta-analysis

**DOI:** 10.1186/s12903-024-04451-y

**Published:** 2024-06-12

**Authors:** George Uchenna Eleje, Emeka Emmanuel Okoh, Emeka Philip Igbodike, Folahanmi Tomiwa Akinsolu, Francisca Obiageri Nwaokorie, Joanne Marie Lusher, Maha El Tantawi, Abideen Olurotimi Salako, Oliver Chukwujekwu Ezechi, Morẹ́nikẹ́ Oluwátóyìn Foláyan

**Affiliations:** 1 Oral Health Initiative, Nigerian Institute of Medical Research, Yaba, Lagos, Nigeria; 2 Department of Obstetrics and Gynaecology, Nnamdi Azikiwe University Teaching Hospital Nnewi, Nnewi, Nigeria; 3 Effective Care Research Unit, Department of Obstetrics and Gynaecology, Nnamdi Azikiwe University, Awka (Nnewi Campus), P.M.B. 5001, Nnewi, Anambra State Nigeria; 4 Department of Community Medicine and Primary Healthcare, Nnamdi Azikiwe University Teaching Hospital Nnewi, Nnewi, Nigeria; 5 Department of Obstetrics and Gynaecology, Havana Specialist Hospital, Surulere Lagos, Nigeria; 6 Department of Obstetrics and Gynaecology, Advanced Minimal Access Surgical Hospital, Kelina Hospital, Victoria Island, Lagos, Nigeria; 7 Nigerian Institute of Medical Research, Yaba, Lagos, Nigeria; 8 Department of Public Health, Faculty of Medicine, Lead City University, Ibadan, Nigeria; 9 Department of Medical Laboratory Science, University of Lagos, Lagos, Nigeria; 10 Provost’s Group, Regent’s University London, London, UK; 11 Department of Pediatric Dentistry and Dental Public Health, Faculty of Dentistry, Alexandria University, Alexandria, 21527 Egypt; 12 Department of Child Dental Health, Obafemi Awolowo University, Ile-Ife, Nigeria

**Keywords:** Nigeria, Cancrum oris, Stomatitis gangrenosa, Case fatality rate, Malnutrition, Measles, Malaria

## Abstract

**Objectives:**

To determine the prevalence, case-fatality rate, and associated risk-factors of Noma in children in Nigeria.

**Methods:**

Search was conducted in PubMed, Google Scholar, and Cochrane Library databases. Data were extraction using a double-blind approach. Discrepancies were resolved by a third reviewer. Heterogeneity was evaluated using I^2^ statistics. Random-effects model was used for the meta-analysis and subgroup analysis was conducted. The study quality was evaluated using standard Critical-Appraisal-Checklist.

**Results:**

Of the 1652 articles identified, 12 studies that met the inclusion criteria included 871 cases of Noma. Two studies had high-risk of bias and were excluded in the meta-analysis. Pooled prevalence of Noma was 2.95% (95%CI:2.19–3.71; Z = 7.60; *p* < 0.00001, I^2^:100.0). Case fatality was reported in one study. Sex-distribution had a male-to-female ratio of 1.1:1. Malnutrition (88.42%, 95%CI:52.84–124.00; I^2^:100.0), measles (40.60%; 95% CI:31.56–49.65; I^2^:100.0) and malaria (30.75%; 95% CI:30.06–31.45; I^2^:100.0) were the most notable associated risk-factors. Prevalence of Noma was non-significantly lower in southern (1.96%,95%CI:1.49–2.44;6 studies) than in northern (4.43%; 95%CI:-0.98-9.83; 4 studies) Nigeria. One study reported the prevalence of Noma in children younger than 5 years.

**Conclusions:**

About every 3 in 100 children in Nigeria had Noma and the prevalence was non-significantly higher in northern than southern Nigeria. Malnutrition, measles, and malaria were major associated risk-factors. Case-fatality rate and prevalence based on different age-groups were inconclusive.

**Supplementary Information:**

The online version contains supplementary material available at 10.1186/s12903-024-04451-y.

## Introduction

Noma, also known as cancrum oris or stomatitis gangrenosa, is referred to as a neglected disease [1–4] with very little written about it despite its destructive effects [5]. It results from bacterial infection (such as *Prevotella sp., Spirochaetes sp., Peptostreptococcus sp., Borrelia vincentii, Fusiformis fusiformis*, and *Fusobacterium necrophorum*) and is associated with poverty [4]. The intraoral pathogenic microorganisms compromise the immune system’s ability to resist infection leading to a rapidly gangrenous infection that spreads to the tissues of the face [6], and ultimately causes the destruction of the cheek, nose, lips and/or the eye lid [7]. This leads to facial disfigurement [8, 9], trismus, oral incontinence, and speech issues [10, 11]. Noma also leads to considerable deterioration in quality of life due to lifelong physical and mental health sequelae [12]. It is also highly fatal [13]: without treatment, 90% of individuals with Noma die within a week or less [10].

The recognized risk factors associated with Noma encompass inadequate oral hygiene, malnutrition, and weakened immune responses due to factors including measles or other illnesses that compromise immunity [6, 14–16]. This situation is compounded by limited access to nourishing food and essential medical care resulting from financial constraints and geographical barriers [17, 18]. Additionally, suboptimal feeding practices, inadequate hygiene and sanitation conditions [19], as well as limited availability of vaccinations contribute to the challenges faced by affected populations [17].

Individuals afflicted by Noma commonly inhabit rural regions, where the prevalence of poverty is higher than urban centers [20, 21]. Within these communities, there is greater susceptibility to concurrent health issues associated with Noma [22], including a higher likelihood of compromised oral health [23]. This is driven by the financial inability to acquire items like toothbrushes and toothpaste, which are essential for oral hygiene [23]. Moreover, children bear the brunt of Noma’s impact, making it a manifestation of poverty and malnutrition among this subgroup of the population [19]. The vulnerability of children to Noma is acknowledged by prominent bodies such as the UN Committee on the Rights of the Child and the Committee on Economic, Social, and Cultural Rights [24, 25]. Therefore, placing a priority on concerted efforts to eliminate Noma would contribute significantly to the realization of the health and well-being rights of the most vulnerable members of society.

Despite the well-documented severe repercussions of Noma, obtaining precise and evidence-based reports about it has proven elusive, and the global extent of its prevalence remains uncertain [26, 27]. Most available reports concerning Noma are case studies [28], originating from Africa and Asia [5, 29–36]. Among these, Nigeria stands out as one of the endemic countries for Noma in Africa [37]. The scarcity of epidemiological information on Noma can be attributed to its prevalence primarily among the most economically disadvantaged segments of society [38]. A systematic review aimed at compiling worldwide Noma data did not incorporate a meta-analysis or provide a consolidated prevalence report [39]. A recent bibliometric analysis that focused on Noma publications substantiated the limited attention that this disease receives on a global scale [40]. A comprehensive scoping review emphasized the necessity for future research to address critical areas such as assessing disease burden and distribution, identifying the mortality rate, uncovering risk factors, and elucidating factors influencing prognosis and post-treatment outcomes [41]. Presently, the World Health Organization officially recognized Noma as a neglected tropical disease on 13th December 2023 following a recommendation of the Strategic and Technical Advisory Group for Neglected Tropical Diseases [1–3].

Consequently, the present study was conceived as a response to the existing knowledge gap regarding Noma. The Nigerian Ministry of Health underlined Noma’s status as a significant national public health concern, emphasizing the urgent need to generate reliable evidence to inform program planning [42]. The primary objectives of this systematic review and meta-analysis were to ascertain the prevalence of Noma, its case fatality rate, and the associated risk factors among children in Nigeria.

## Methods

### Study protocol

The study was performed according to an a priori defined protocol for systematic review and meta-analysis, with PROSPERO number: **CRD42023396391.** The whole study was reported following the Preferred Reporting Item for Systematic Reviews and Meta-analyses (PRISMA) statement and checklist [43]. Each review stage was performed by two authors in a blinded fashion, and disagreements were solved by a discussion with a third author.

### Search strategy

Multiple searches were conducted using electronic databases, including PUBMED, Google Scholar, and The Cochrane Library, covering the period from their inception to July 2023. The following search terminologies were used in the search strategy subsection: epidemiology OR prevalence AND Noma OR Cancrum Oris OR Oris OR Cancrum OR stomatitis gangrenosa AND malnutrition OR malnourished OR poverty OR Acquired Immune Deficiency Syndrome OR Human Immunodeficiency Virus OR malaria OR measles OR Chicken pox AND ulcerative gingivitis OR infections OR oro-facial gangrenous infection AND Children OR child OR under-five OR adolescent OR Chil* OR infant* AND Nigeria OR Sub-sahara* Africa*. The initial search syntax was developed for PubMed and later adapted to fulfil the unique search criteria of the other databases, as detailed in Supplemental File 1. The review process involved evaluating the titles and abstracts of all the references obtained from eligible articles. Supplementary articles were also discovered by examining the reference lists of already identified articles.

### Study selection

Two researchers (GUE and EEO) independently determined and selected the studies to be included in the review considering the inclusion and exclusion criteria. The titles and abstracts of all studies were screened, followed by assessment of full texts of selected studies in detail to determine eligibility. The articles selected independently by the two authors were compared. A joint decision was reached by meeting with the third author (EPI) about the articles on which there was a disagreement.

### Inclusion and exclusion criteria

Both published and unpublished studies were included in this systematic review. In cases where a study was reported in multiple sources, the most comprehensive and current version was selected. To be considered eligible, studies needed to fall under the following categories: cross-sectional studies, cohort studies, and case-control studies. Additionally, studies were included if they presented data in children population (0 to 16 years), and available data for at least one of the primary outcomes.

Exclusion criteria encompassed studies focused on adult populations, studies that did not provide information on prevalence of Noma among children in Nigeria, studies lacking sample size details, studies conducted outside Nigeria, studies with inaccurate or unavailable outcome data, and studies featuring duplicate samples. Furthermore, review articles were omitted from consideration. Studies with overlapping data from other included studies, along with case reports, case series, or editorials, were also excluded. Language restrictions were not imposed. Two authors independently assessed each study chosen for inclusion in the research, and a third author cross-checked the evaluation.

### Quality and risk of bias within studies assessment

Evaluations of the risk of bias followed an adapted version of the observational studies’ risk of bias tool developed by Hoy and colleagues [44]. Each study was scrutinized for nine risk of bias domains namely: alignment of the study’s target population with the national population in terms of pertinent variables, the congruence between the sampling frame and the target population, the utilization of random selection techniques in the sampling process, the minimization of non-response bias through a substantial response rate, the direct collection of data from participants rather than proxies, the precision of the study’s case definition, the reliability and validity of the instrument employed for data collection, the consistency in the mode of data collection across all participants, and the accurate description of the numerator and denominator for the parameter of interest.

To rate each specific parameter, the authors reached a consensus to assign a score of 0 if the study met the criterion and 1 if it did not. Subsequently, a composite quality index was calculated, and the risk of bias was categorized as low (0 to 3), moderate (4 to 6), or high (7 to 9), and reported in the Supplementary File 2. Only articles with low and moderate risk of bias were included in the meta-analysis. Two authors independently evaluated each study against the critical appraisal checklist, with the third author providing verification.

### Outcome measurement

The outcome measures for this systematic review and meta-analysis encompassed the prevalence of Noma in Nigeria and the case fatality rate of Noma in Nigeria. The associated risk factors were also outcomes of interest. Prevalence of Noma in children was the number of children (0–16 years) with the disease divided by the number of children in the defined study population [45]. These measures were intended to be quantified through the application of meta-analysis techniques.

### Subgroup and sensitivity analyses

Subgroup analyses were carried out to uncover potential sources of heterogeneity. Some subgroups were categorized based on the age brackets in the included studies, i.e., 0–5 years and 6–16 years. The proportion (95% CI) of children with Noma in Nigeria was divided into strata based on publication years (1970–1999 vs. 2000–2023), Nigerian regions (south vs. north), and the age distribution of the studied population (0–5 years vs. 6–16 years). All subgroup analyses were performed on a study level. A subgroup effect was considered present when the interaction test in Review Manager 5.4.1 indicated group differences (*p* < 0.10). Sensitivity analysis was conducted to investigate the impact of risk of bias (high risk of bias versus low risk of bias) on prevalence rate and case fatality rate. We also performed a leave-one-out sensitivity analysis to reveal the influence of individual studies on the overall pooled prevalence from all studies.

### Assessment for publication bias

Publication bias and the potential impact of small studies were evaluated employing funnel plots and Egger’s tests for outcomes encompassing at least ten studies [46].

### Data extraction

A standardized data extraction form was devised by the researchers to uniformly collect information from each study that was incorporated into the systematic review. This data extraction form encompassed various details, including the first author’s name, year of publication, the year when data collection occurred, the political region where the study was conducted, the city where the study was conducted, the study setting, the study design, the count of cases, the mean or median age of the included children, and the prevalence, the sample size, the tools employed for data collection, the gender distribution among cases, and the stage of Noma. According to the World Health Organization, Noma can be classified into five stages: Stage 1: acute necrotising ulcerative gingivitis; Stage 2: oedema; Stage 3: gangrene; Stage 4: scarring; and Stage 5: sequel [21]. Where datum was presented in percentage form, the number of events was derived from the total number of participants in the respective group. Furthermore, the authors sought any missing information directly from the original authors via email if feasible.

### Data synthesis and analysis

The analysis was conducted using Review Manager 5.4.1 (Copenhagen: The Nordic Cochrane Centre, Cochrane Collaboration, 2021). The prevalence of Noma was calculated for each individual study and subsequently pooled to derive an overall estimate. These prevalence values were visually presented on a forest plot. The DerSimonian-Laird random effects model was employed across all analyses. Furthermore, available information on the case fatality rate, associated risk factors, were documented from the included studies.

The prevalence of Noma within the study population was combined, employing the percentage (along with a 95% confidence interval) as the effect size. This combined effect was calculated using the generic inverse variance (IV) approach, as the effect of a single rate and its standard error closely resembles the Rate Difference (RD). To assess study heterogeneity, the Q test and forest plots were utilized. The extent of statistical heterogeneity among studies was assessed through the inconsistency index I^2^ (Higgins *I*^2^), with categorizations of null (I^2^ = 0), insignificant (0 < I^2^ ≤ 25%), low (25 < I^2^ ≤ 50%), moderate (50 < I^2^ ≤ 75%), and high (I^2^ > 75%) [47].

].

## Results

### Search results and study selections

**Fig. 1:**
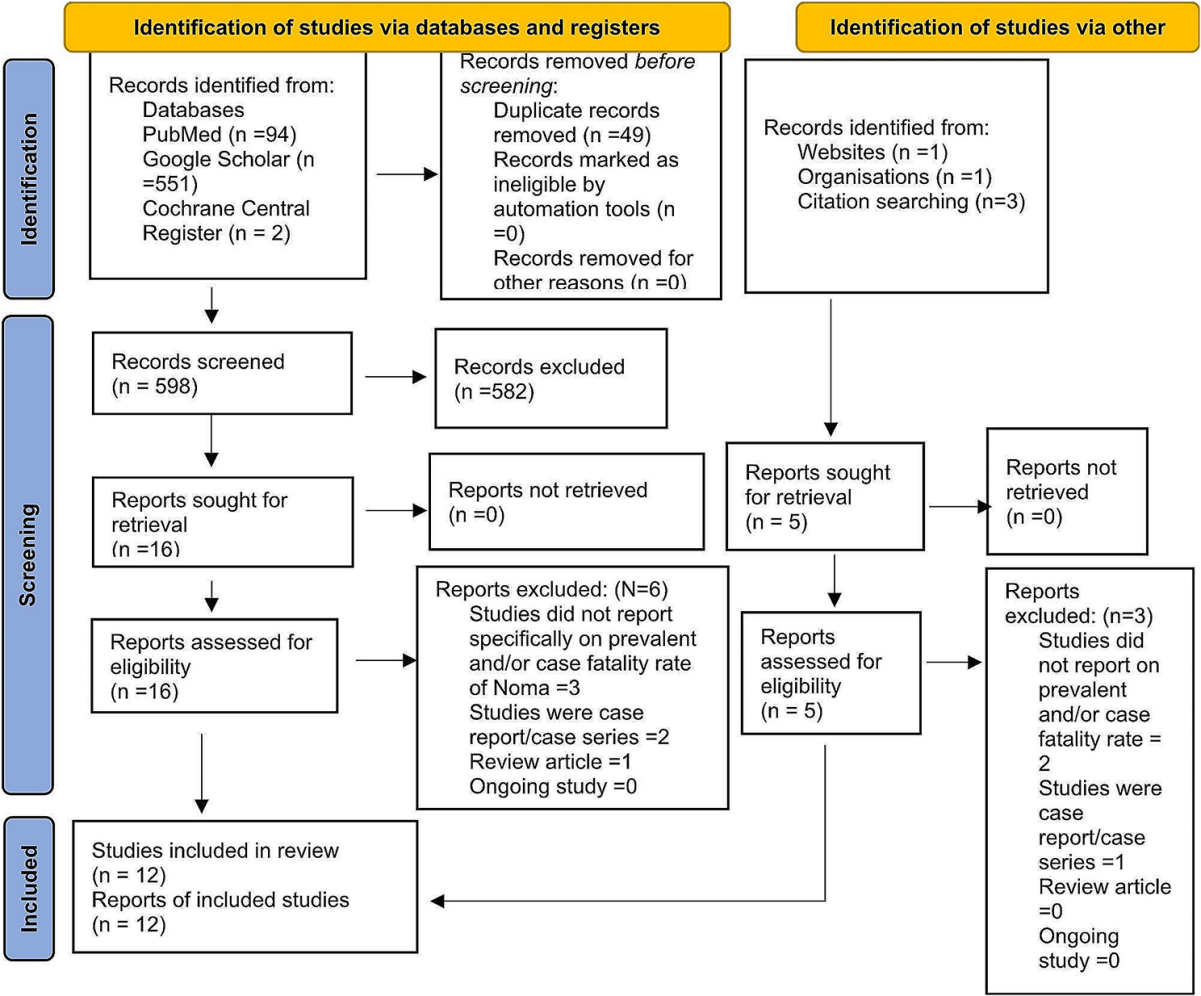
PRISMA flow chart

Figure 1 displays the PRISMA flow diagram of literature search and selection. The database searches, hand searches and other sources’ search yielded 652 resources. A total of 49 duplicates were removed, leaving 603 articles for title and abstract screening. After title and abstract scanning, 12 articles were considered relevant. The full manuscripts were reviewed for inclusion [48–59] and the extracted data are shown in Tables 1 and 2.

**Table 1:** The characteristics of the included studies

SN	First Author (Year of publication)	Period of participants’ recruitment	Region of recruitment	Study Setting or location	City/Area	Study Design	No of noma cases	Sample Size	Mean or median age	Prevalence
1	Adeniyi (2019) [48]	Jan 1999 to December 2011	Northwest	Noma Children Hospital, Sokoto	Sokoto	Retrospective Cross-sectional	159	1923	3.0 ± 1.4 years	8.3%
2	Bello (2019) [49]	2010 to 2018	North Central	Cleft and Facial deformity, an NGO	Abuja	Retrospective Cross-sectional	10	78	2.7 years, 8.4 years	12.8%
3	Denloye (2003) [50]	1986 to 2000	Southwest	Ibadan University College Hospital, Dental Centre	Ibadan	Retrospective Cross-sectional	45	6390	4.2 +/- 2.7 years	0.70%
4	Enwonwu (1972) [51]	1963 to 1965	South	Multi-Centres	Multisite	Cross-sectional	69	1068	Not stated	6.4%
5	Farley (2020) [52]	September 17 to November 5, 2018	Northwest	Kebbi and Sokoto state	Kebbi and Sokoto	Cross-sectional	237	62	Not stated	3.30%
6	Fierger (2003) [53]	October 1996 to September 2001	Northwest and North south	Sokoto state specialist hospital	Sokoto	Not reported	5	341	Not stated	0.50%
7	Fomete(2018) [54]	2006 to 2014	Northwest	Ahmadu Bello University Teaching Hospital	Zaria	Retrospective Cross-sectional	5	89	Not stated	5.60%
8	Idigbe (1999)* [55] a. Idigbe (1999) southern Nigeria b. Idigbe (1999) northern Nigeria	October 1996 to April 1998	a. Southsouth b. Northwest	Multicentre	a. Lagos, Kwara, Ondo, Ogun, Osun and Oyo. b. Sokoto, Zamfara and Kebbi.	Cross-sectional	a. 10 b. 129	a. 8.9 million b. 3.5 million	Not stated	a. 0.0001% b. 0.003%
9	Oginni (1999) [56]	1982 to 1996	Southwest, Nigeria	Obafemi Awolowo University Teaching Hospital	Ile-Ife	Retrospective Cross-sectional	142	8481	4.65 ± 2.57 years	1.70%
10	Osuji (1990) [57]	Not stated	Southwest	Ibadan University College Hospital, Dental Centre	Ibadan	Cross-sectional	5	1359	Not stated	0.37%
11	Otuyemi(1992) [58]	Not stated	Southwest	Obafemi Awolowo University Teaching Hospital	Ile-Ife	Cross-sectional	10	633	Not stated	1.58%
12	Otuyemi (1998) [59]	Not stated	Southwest	Obafemi Awolowo University Teaching Hospital	Ile-Ife	Cross-sectional	25	2462	Not stated	1.02%

*Idigbe (1999) one article reporting two independent data for northern and southern Nigeria

**Table 2:** The characteristics of the included studies II

SN	First Author (Year of publication)	Number of males	Number of females	Quality score	Risk of bias
1	Adeniyi (2019) [48]	NR	NR	1	Low
2	Bello (2019) [49]	6	4	7	High
3	Denloye (2003) [50]	17	17	1	Low
4	Enwonwu (1972) [51]	NR	NR	1	Low
5	Farley (2020) [52]	NR	NR	3	Low
6	Fierger (2003) [53]	NR	NR	1	Low
7	Fomete (2018) [54]	2	3	1	Low
8	Idigbe (1999) [55]	43	43	7	High
9	Oginni (1999) [56]	70	72	4	Moderate
10	Osuji (1990) [57]	3	2	1	Low
11	Otuyemi(1992) [58]	NR	NR	1	Low
12	Otuyemi (1998) [560]	17	8	1	Low

NR = Not reported. All included studies used clinical methods for data collection and diagnosis

Nine articles [60–68] were excluded after full text screening, with reasons (such as review article, case report or case series) highlighted in Supplementary File 3 (Appendix 3).

### Characteristics of the included studies

The characteristics of each study are detailed in Table 1. The publication timeline of these studies encompasses the years 1972 through 2020. The sample size exhibited variability, ranging from 62 to 8,900,000 individuals, with a total of 12,420,584 across the entirety of the studies. The diagnosis of Noma in all studies was predicated upon clinical assessment.

Out of the 12 studies, five (41.7%) obtained their data from the northern states of Nigeria [48, 49, 52–54], and six (50.0%) collected data from the southern states only [51, 56–59]. One study (8.3%) separately presented data for the northern and southern regions within a single report [55].

Every study included in the analysis presented information either about Noma prevalence or its estimation. One study [56] reported the case fatality rates, and five studies examined the associated risk factors [48, 49, 51, 57, 59]. Additionally, one study offered insights into the staging of Noma [56], while another provided information on the prevalence for participants aged under 5 years and those aged between 6 and 16 years [53].

### Prevalence of noma in Nigerian children

**Fig. 2:**
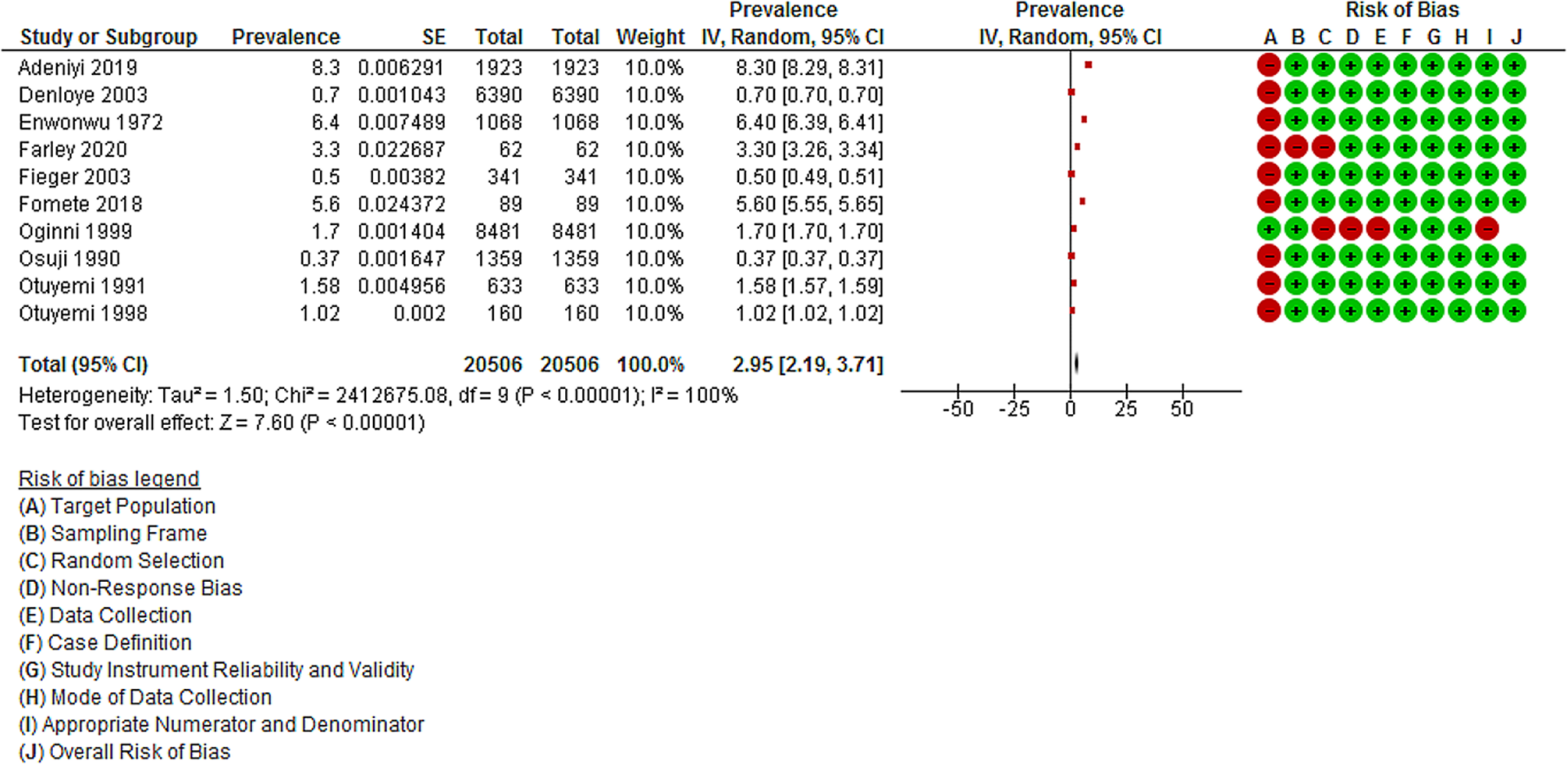
Meta-analysis showing the pooled prevalence of noma in nigerian children. *Abbreviation* IV = inverse variance; Red circle = High risk of bias; white or empty circle = Moderate risk of bias; Green circle = Low risk of bias

The pooled prevalence of Noma in Nigerian Children or its estimation as reported by the included studies [48–59] was 2.95% (95% CI: 2.19–3.71, Z = 7.60, *p* = 0.00001, I^2^: 100.0) as shown in Fig. 2.

### Case fatality rate of Noma

Only one [56], out of the 12 included studies, reported a case fatality rate of Noma at 0%

### Frequency of Noma risk factors

**Fig. 3:**
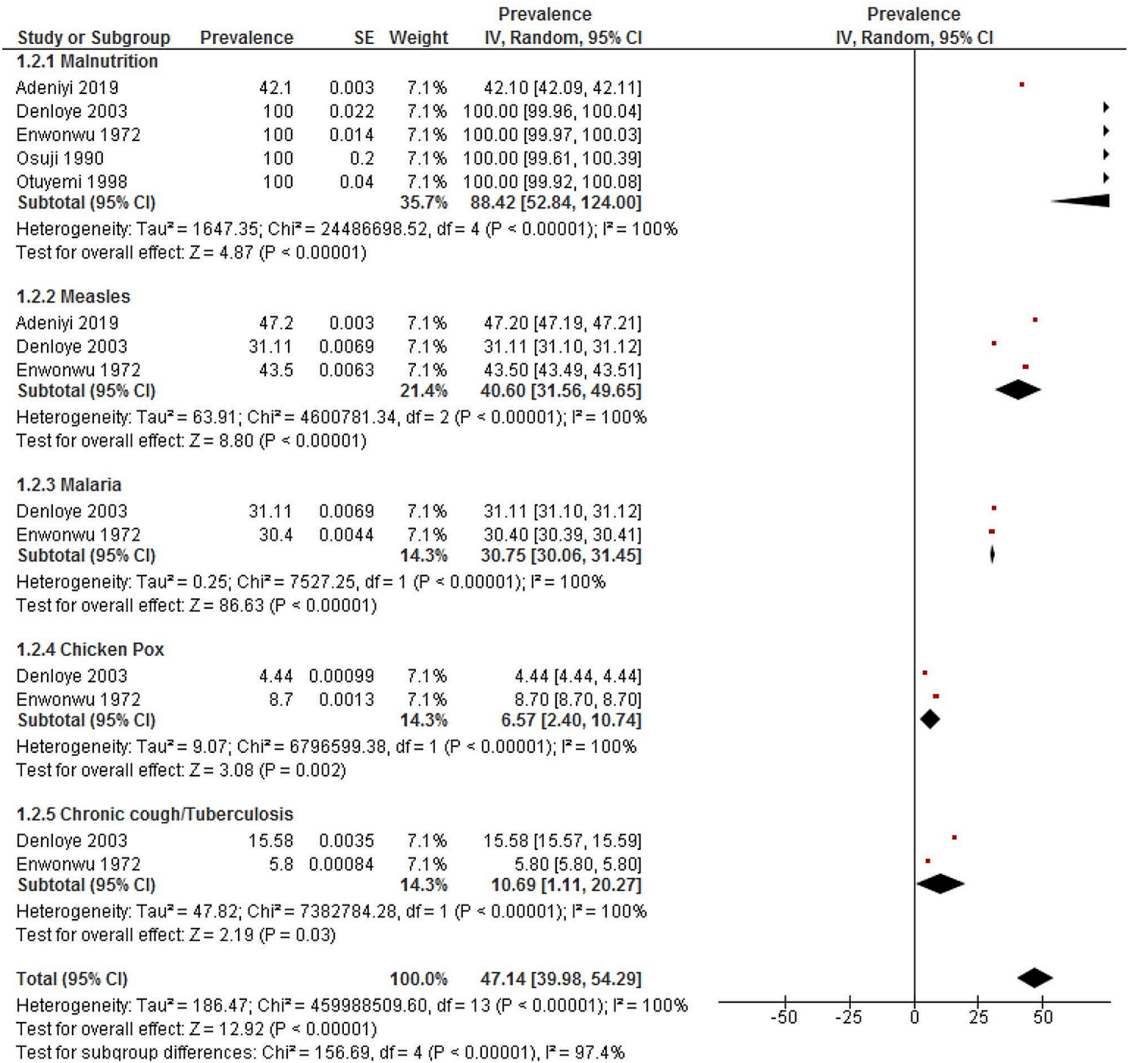
Meta-analysis of frequency of Noma associated risk factor in Nigeria. *Abbreviation* IV = inverse variance

Five of the 12 studies reported on the associated risk factors [48, 50, 51, 57, 59]. The highest prevalence was for malnutrition (88.42%, 95% CI: 52.84–124.00, I^2^: 100.0), followed by measles (40.60%, 95% CI: 31.56–49.65, I^2^: 100.0) and malaria (30.75%, 95% CI: 30.06–31.45, I^2^: 100.0). This is shown in Fig. 3.

### Stages of Noma

Three [51, 52, 56] studies reported on the stage of Noma. One [56] study reported the actual stages as follows: stage 1 (2.3%), stage 2 (39.7%), stage 3 (42.0%), stage 4 (13/0%). Another study [51] reported that advanced cases of Noma were the types frequently encountered and presented hardly any possibility of confusion with other lesions. In Farley et al. study [52], no cases of late stage Noma were detected.

### Gender distribution of Noma cases

Seven studies [49, 50, 54–57, 59] specifically addressed the gender distribution of Noma cases. The cumulative number of cases reported in the seven studies was 169 males and 149 females, resulting in a male-to-female ratio of 1.1:1.

### Quality assessment and Risk of Bias

The comprehensive results quality assessment are in Supplemental file 2 and Fig. 2. Among these, nine (75.0%) studies were categorized as high quality [49–54, 57–59], while one (8.3%) had moderate quality [56]. Two (16.7%) studies had low-quality as they had high risk of bias [49, 55] and were excluded for the meta-analysis. The other 10 studies were retained for synthesis of knowledge.

### Subgroup analysis

#### Year of publication

**Fig. 4:**
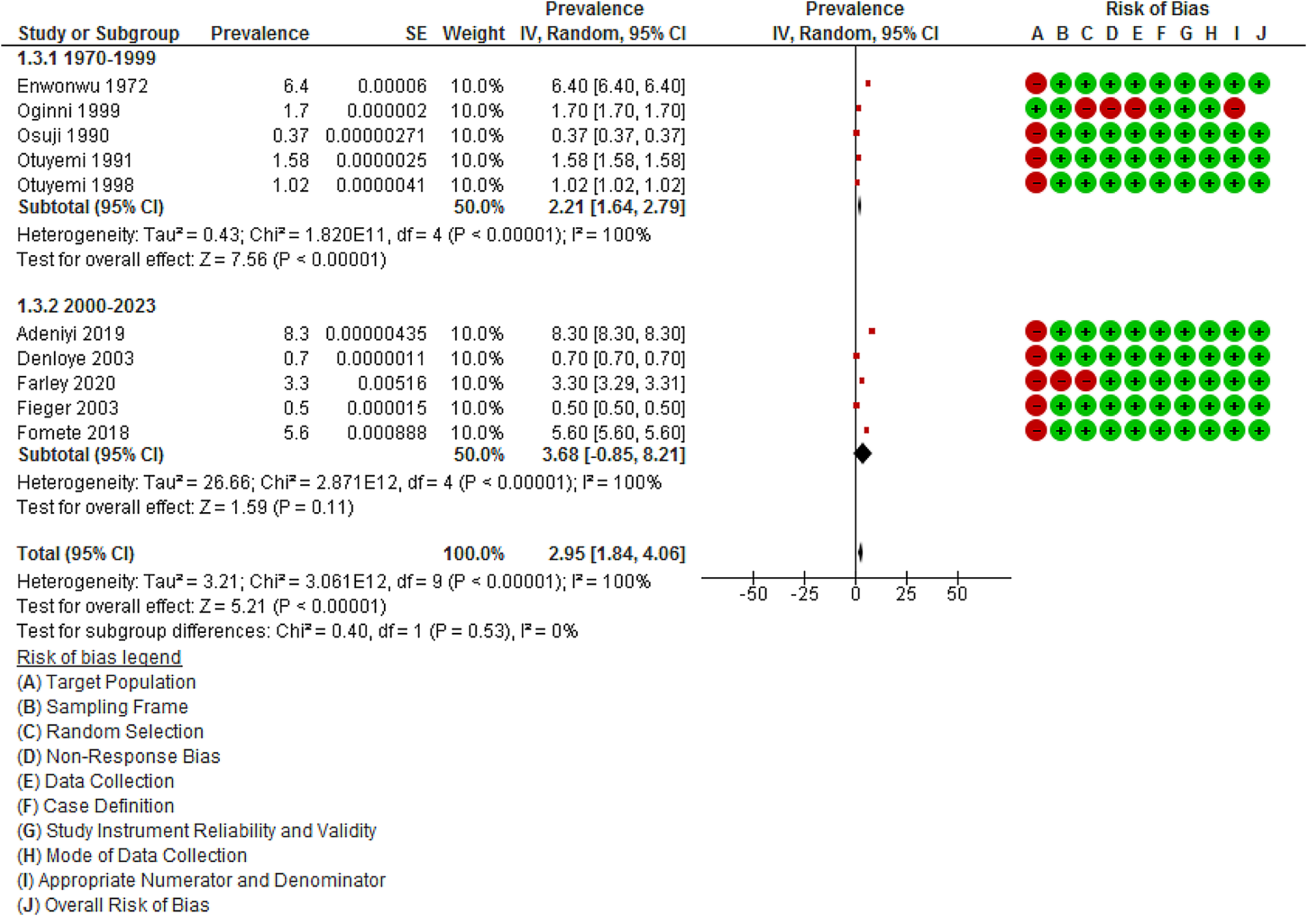
Meta-analysis showing the pooled prevalence of Noma in Nigeria according to year of publication. *Abbreviation* IV = inverse variance

The studies were categorized into two time periods: from 1970 to 1999, and from 2000 to 2023. The prevalence in five studies [51, 56–59] published between 1970 and 1999 (2.21%; 95% CI: 1.64 to 2.79%, *p* < 0.00001; I^2^ = 100.0%; 5 studies) was lower than the prevalence in five studies [48, 50, 52–54] published between 2000 and 2023 (3.68%; 95% CI: -0.85 to 8.21%, *p* < 0.00001; I^2^ = 100.0%; 5 studies), Fig. 4. Test for subgroup differences showed no significant difference (*p* = 0.53). Notably, the level of heterogeneity remained consistent, suggesting that the variation in prevalence is not attributed to the year of publication (I^2^ = 100.0% vs. 100.0%).

#### Region of study

**Fig. 5:**
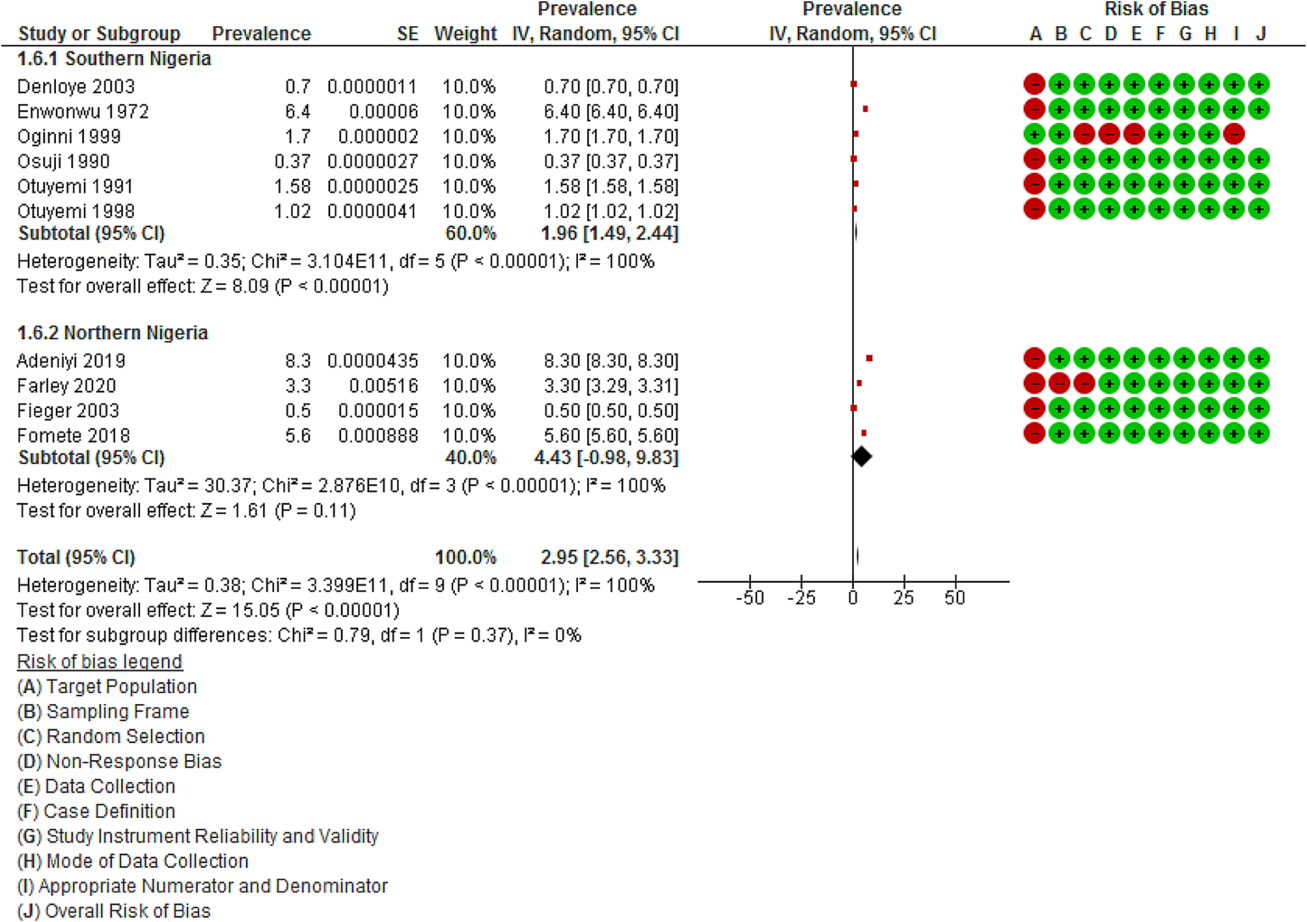
Meta-analysis of prevalence of Noma in Nigerian Children according to regions of Nigeria. *Abbreviation* IV = inverse variance

The prevalence of Noma in southern Nigeria in six studies [50, 51, 56–59] (1.96%; 95% CI: 1.49 to 2.44%, *p* < 0.001; I^2^ = 100.0%) was lower than in northern Nigeria [48, 52–54] (4.43%; 95% CI: -0.98 to 9.83%, *p* < 0.00001; I^2^ = 100.0%) as shown in Fig. 5. Notably, the tests for subgroup differences indicated no significant difference (*p* = 0.37, I^2^ = 0.0%). The level of heterogeneity remained consistent, suggesting that the variation in prevalence is not attributed to the region of study (I^2^ = 100.0% vs. 100.0%).

#### Age of study population

Only one study [53] reported the prevalence according to age 0–5 years (0.50%; 95% CI: 0.50 to 0.50%) and 6–16 years (0.50%; 95% CI: 0.50 to 0.50%). Nine studies [48, 50–52, 54, 56–59] reported the prevalence of Noma for the age group 0–16 years.

### Other pre-planned subgroup analyses

Other pre-planned subgroup analyses (early-stage vs late stage Noma and HIV positive vs HIV negative) were not done because they were not available in the included studies.

### Sensitivity analysis

**Fig. 6:**
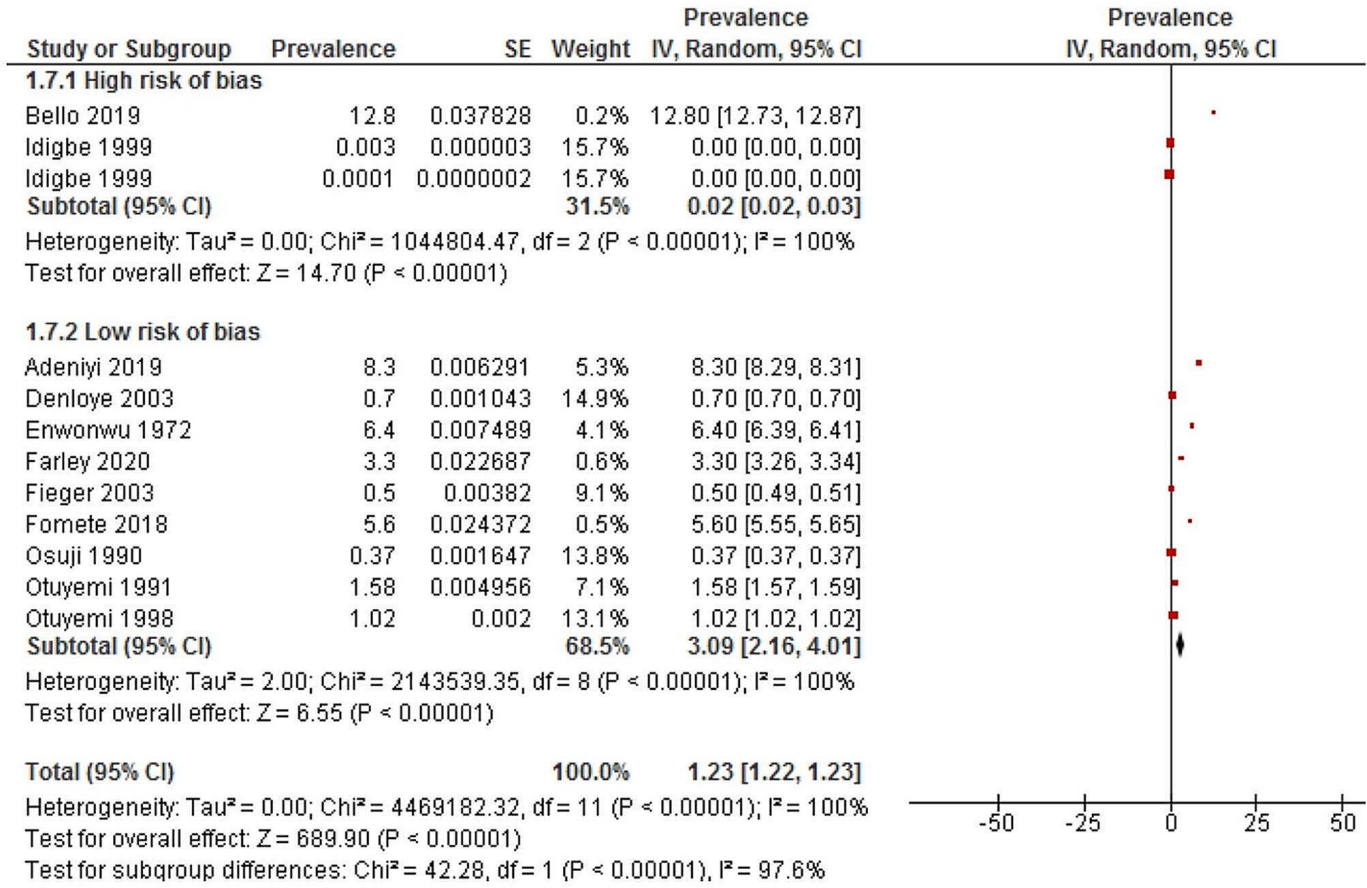
Sensitivity analysis showing the prevalence of Noma in Nigerian children according to the pooled estimate of studies with high and low risk of bias. *Abbreviation* IV = inverse variance

**Fig. 7:**
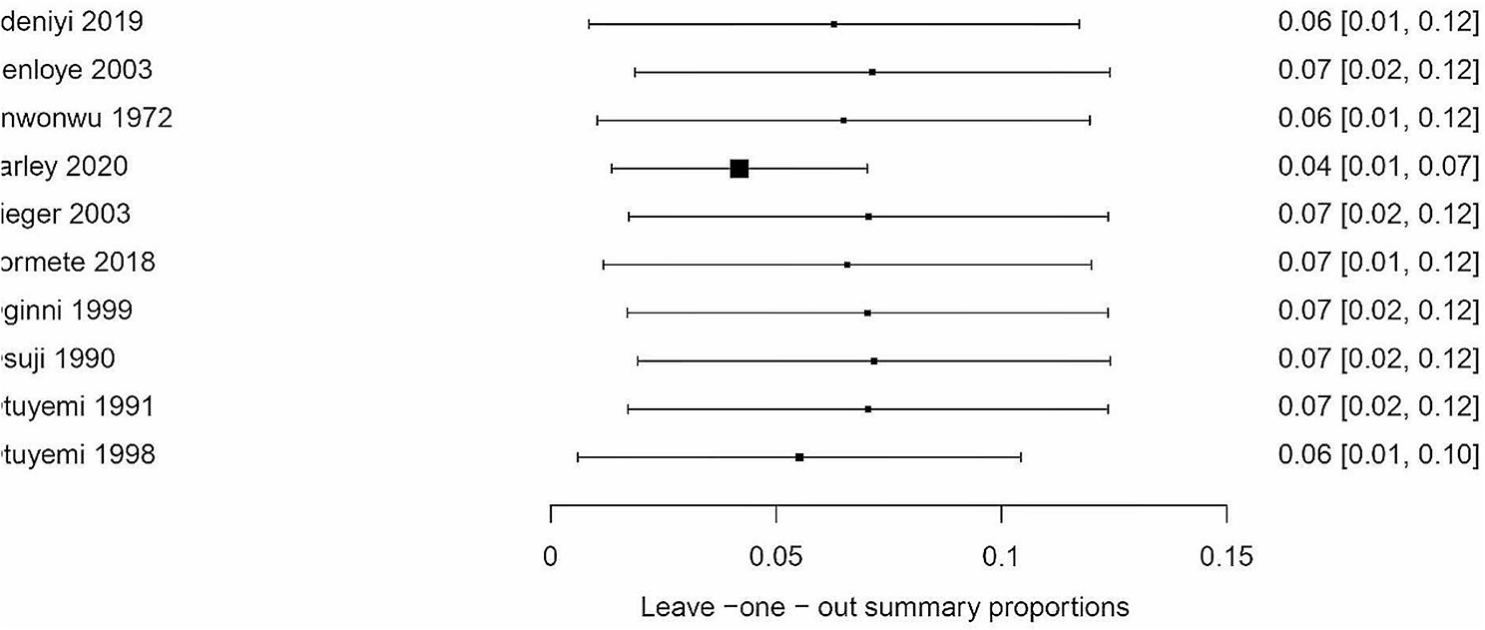
Leave-one-out sensitivity analysis showing the influence of individual studies on the overall pooled prevalence of Noma from all the studies

**Fig. 8:**
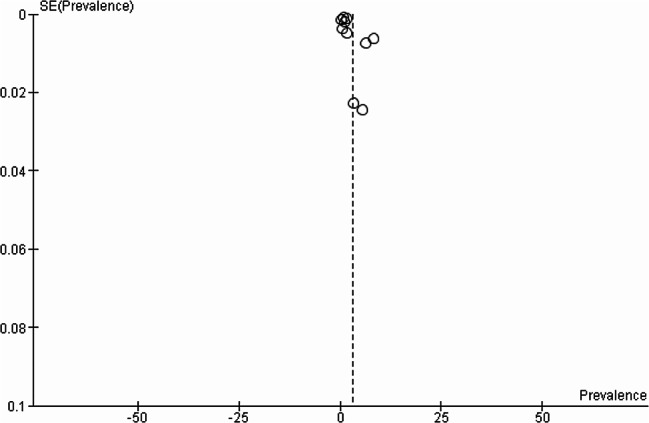
Funnel plot of the included studies

Sensitivity analysis was conducted using a random-effects model. Studies with high risk of bias [49, 55] had a significantly lower prevalence of Noma (0.02%; 95% CI 0.02–0.03; *I*^2^ =100%; *p* <0.00001) than studies with low risk of bias [48, 50–54, 57–59] (3.09%; 95% CI 2.16 to 4.01; *I*^2^  =100%; *p<0.00001).* Using the leave-one-out sensitivity analysis, the result of a random effect model revealed that, the pooled prevalence of Noma among Nigerian children was not influenced by a single study (Figs. 6, 7 and 8). We could not perform sensitivity analysis according to case fatality rate because only one study reported this [56].

### Publication bias assessment

**Fig. 9:**
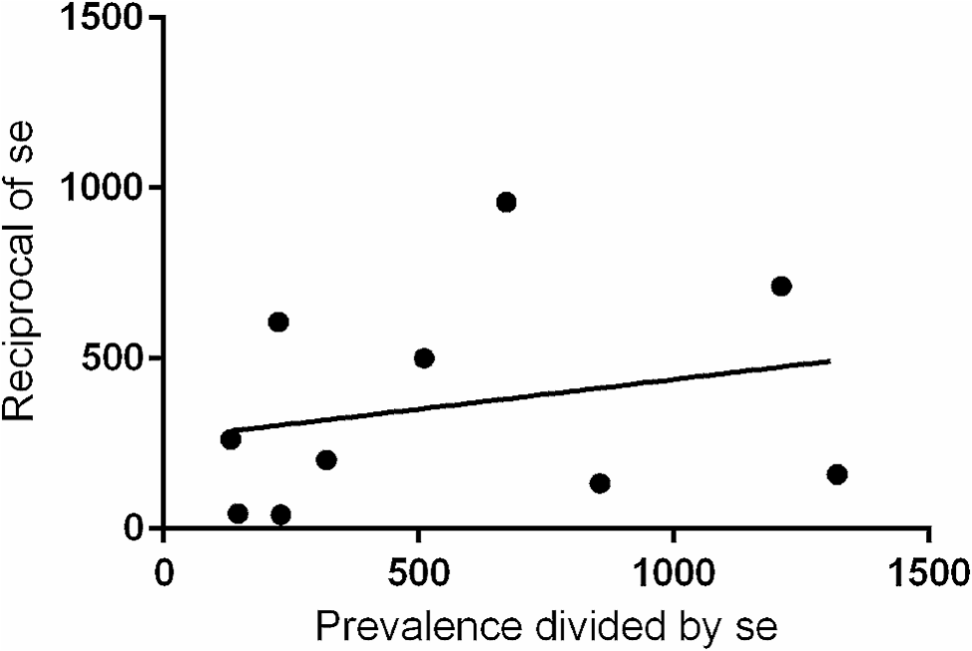
Egger test* regression graph of included studies. *Abbreviation* se = standard error; *Data: number of XY pairs: 10; equation: y = 0.0.1745X + 264.0; Best-fit values: Slope: 0.1745 ± 0.2456; Y-intercept: 264.0 ± 171.7; X-intercept: -1513; 1/slope: 5.732; Goodness of fit: R square: 0.05934; Sy.x: 323.7; F: 0.5046; DFn, DFd: 1.8; p-value: 0.4977 (not significant)

There was no evidence of asymmetry in the funnel plot in Fig. 8, suggestive that no significant publication bias exists. The Egger test (Fig. 9) was used as a statistical method to assess publication bias; the effects of research on the H0 test are not significant (Egger test, *p* = 0.4977).There was also no evidence of small study effects (as indicated by the Eggers test (Fig. 8) which was not significant.

### Patient and public involvement

There was no patient or public involvement in the design or execution of this systematic review and meta-analysis.

## Discussion

This study was the first meta-analysis to evaluate the cumulative and overall prevalence of Noma in children in Nigeria. This systematic review and meta-analysis indicates that approximately 3 in 100 children in Nigeria have Noma. The prevalence increased non-significantly from 2.21% between 1970 and 1999 to 3.68% between 2000 and 2023. The prevalence in southern Nigeria was non-significantly lower than that in northern Nigeria. In addition, out of every 100 children with Noma, about 88 children were malnourished, 40 had measles and about 31 had malaria. This current review has a number of insights.

Firstly, the prevalence of Noma in Nigerian children raises a concern, particularly considering the possibility of its elimination [69, 70]. Secondly, the higher prevalence of Noma in Northern Nigeria appears to correlate with higher poverty levels in the region [71], inadequate access to medical care [72], severe malnutrition [73], compromised access to safe drinking water, substandard sanitation practices [74], and suboptimal vaccination coverage, especially poor vaccination against measles, which is a risk factor for Noma [75]. Thirdly, there is evidence of a rise in Noma prevalence, possibly linked to economic downturns exacerbating child malnutrition in Nigeria [76]. On the contrary, it is possible that the increase in the prevalence could also be attributed to a ‘harvesting’ phenomenon due to improved healthcare seeking behavior by patients with Noma, improved access to Noma-specialized care, enhanced diagnostic proficiency, or increased public awareness about Noma. Previously, inadequate diagnosis by healthcare personnel led to child fatalities from Noma [4].

These findings emphasize a critical need to address Noma as a neglected tropical disease, demanding investment for its eradication [1–4]. A particular emphasis should be placed on Northern Nigeria to advance the goal of eliminating Noma from the country. However, developing a comprehensive agenda for Noma elimination necessitates a multifaceted approach that effectively targets recognized risk factors like malnutrition, measles, and malaria. This is further underscored by the findings of this study and a preceding report [77], which highlighted malnutrition as the predominant risk factor for Noma. Malnutrition and measles increase the vulnerability of the oral mucosa to opportunistic pathogens, by inducing prolonged impairment of acquired immunological memory, and rendering individuals more susceptible to bacterial and viral pathogens [78]. Measles and malaria are established predisposing factors for Noma [18, 79, 80], often occurring during children’s weaning period and exerting pronounced immunosuppressive effects that elevate the risk of malnutrition [81, 82]. In the absence of a comprehensive strategy for Noma elimination, non-governmental organizations like the Noma Initiative [83–85], Médecins Sans Frontières [84, 85], and Sokoto Noma Hospital [85], which provide care for patients with Noma in Nigeria, will persistently encounter cases stemming from inadequate implementation of systemic strategies to mitigate the risk factors.

While HIV infection is widely recognized as a definite risk factor for Noma [86], this review did not identify any case of HIV-associated Noma. Individuals living with HIV are immunocompromised, elevating their susceptibility to Noma. A solitary study that screened Noma patients for HIV indicated the child had a negative HIV status [87] and the role of HIV as a risk factor from this single case. Nevertheless, the possibility exists that there is an association between Noma and HIV infection as prior cases had been reported in children and adults [88]. It is imperative that further research be conducted to establish whether HIV infection truly constitutes a risk factor for Noma in children in Nigeria, and to understand the pathophysiological mechanisms because of the high burden of children living with HIV in Nigeria [89].

No age or gender disparities for Noma was identified in this review. Previous studies have noted sex-related disparities in childhood infectious diseases due to the influence of sex hormones on the T-helper 1/T-helper 2 cytokine balance, resulting in a heightened vulnerability of males to more severe forms of numerous infections [90, 91]. Symptoms, and disease severity also often vary between sexes and across different age groups [91].

In addition, we found no case fatality, aligning with earlier research that indicates a decline in Noma-related mortality due to the availability of modern antibiotics [92]. It could be that those who died never visited the hospital and their mortality was never accounted for. However, while antibiotics provide a partial solution, they do not fully address the functional, aesthetic, and psychological challenges resulting from the deterioration of soft and hard tissues [93]. The aesthetic issues are particularly noteworthy, given that a significant number of patients are diagnosed in advanced stages, as highlighted by our review. Treating survivors of late-stage Noma remains intricate and resource-intensive, often resulting in outcomes that fall short of achieving optimal functional and aesthetic restoration. Therefore, prioritizing prevention and integrating initiatives into existing healthcare programs becomes crucial [93]. These findings underscore the necessity of further research into these negative aspects revealed by the study.

This review brings attention to the fact that Noma is not an uncommon occurrence in Nigeria, yet it continues to be overlooked by the government and health authorities despite its preventable nature. The national Noma policy should prioritize the promotion of prevention programs that can be seamlessly incorporated into existing funded initiatives aimed at preventing malnutrition, measles, and malaria [42], thereby fostering an integrated approach instead of isolated efforts for Noma eradication. This approach holds the potential for cost-effective programming. Further studies are warranted to delve into the implementation of integrated programs for children that effectively eliminate risks and enable timely access to treatment before the disease reaches its advanced stages.

One of the strengths of this review is the diversity of the population, as this study spans six decades and a wide range of participants. This allows for multiple sub-analyses. It was, therefore, able to explore outcomes that have not been assessed by previous systematic reviews, such as case fatality rates, associated risk factors, gender, and age disparities. Moreover, this systematic review and meta-analysis included studies with high quality scores, thereby bolstering reliable analyses.

This study had some limitations. One was the few numbers of relevant studies have limited the opportunity to conduct sub-group analysis for differences of Noma prevalence by age, disease stage, and the HIV status. We were unable to combine individual odds or risk ratios along with their 95% confidence intervals to assess the risk factors associated with Noma because the relevant data were not provided by the included studies. We could not calculate odds ratios, which measure associations, because there was no reported frequencies of associated risk factors in children without Noma. Additionally, two studies were excluded from our meta-analysis due to inconsistencies in how they assessed Noma prevalence, indicating a high risk of bias [49, 55]. Although our systematic review and meta-analysis focused primarily on the Nigerian population, it is worth noting that studies labeled as originating from the southern region were all conducted in southwestern Nigeria, without representation from the southeastern or south-southern areas. Similarly, studies from the northern region, except one from Abuja [49], were mostly carried out in a few Northwestern states such as Zamfara, Kebbi, and Sokoto. Notably, no studies were found from the Northeast, South-South, and Southeast regions of Nigeria. Only one out of the 12 studies included reported on mortality, with a rate of 0%, which doesn’t accurately reflect the known case fatality reported by the WHO. Moreover, the cross-sectional design of the included studies is also a recognized limitation. For our meta-analysis, we used Review Manager 5.4.1 software, which only supports the default DerSimonian and Laird random-effect meta-analysis. We were unable to conduct sensitivity analyses using restricted maximum likelihood (REML), maximum likelihood (ML), or Paule-Mandel (PM) methods within this software interface. Despite these limitations, this review has generated some new and insightful findings as enumerated in the onset of this discussion.

## Conclusions

The current findings reveal that the combined prevalence of Noma among Nigerian children signifies there is a substantial burden, particularly in Northern Nigeria. Notably, malnutrition, measles, and malaria are risk factors. An effective strategy for Noma elimination in Nigeria should consider economically efficient integrated approaches that target the reduction of malnutrition, measles, and malaria. Further primary investigations are imperative to explore the case fatality rate of Noma particularly for children under the age of five.

### Electronic supplementary material

Below is the link to the electronic supplementary material.

Supplementary Material 1

## Data Availability

All datasets generated and analysed, including the study protocol, search strategy, list of included and excluded studies, data extracted, analysis plans, and quality assessment, are available in the article and upon request from the corresponding author.

## Abbreviations

HIV: Human immunodeficiency virus
PRISMA: Preferred Reporting Items for Systematic Reviews and Meta-Analyses
WHO: World Health Organization
