# Supplementary material for: Prevalence and associated risk factors for noma in Nigerian children: a systematic review and meta-analysis
Source: BMC Oral Health. 2024 Jun 12;24:685. doi: 10.1186/s12903-024-04451-y (PMC11170919; doi:10.1186/s12903-024-04451-y)
Supplement: Supplementary file 1 — Supplementary Material 1 [file 12903_2024_4451_MOESM1_ESM.docx]

**Supplemental File 1:**

**Appendix 1: Pubmed Search Strategy**

1. ("epidemiology"[Subheading] OR "epidemiology"[ tiab] OR "prevalence"[tiab] OR "prevalence"[MeSH Terms])
2. Noma [mh] OR Cancrum Oris [mh: noexp] OR Oris [mh] OR Cancrum [mh] OR stomatitis gangrenosa [tiab] OR NOMA [ tiab]
3. Malnutrition [mh] OR malnurished [mh] OR poverty [tiab] OR AIDS [tiab] OR “Acquired Immune Deficiency Syndrome” [tiab] OR “Acquired Immunodeficiency Syndrome” [tiab] OR Human Immunodeficiency Virus* [tiab] OR Malaria [tiab] OR measles [tiab] OR Chicken pox [tiab]
4. ("ulcerative gingivitis"[MeSH Terms] OR "noma therapy"[tiab] OR ("noma epidemiology"[ tiab]
5. "infections"[ tiab]) OR " oro-facial gangrenous infection "[ tiab])
6. children [mh] OR child [mh] OR under-five [mh] OR adolescent [mh] OR Chil* [tiab] OR infant* [tiab]
7. (neglected disease [tiab] OR noma etiology [tiab] OR oral hygienel [tiab]) AND (neglected diseas* [tiab] OR infection* [tiab])
8. Tropical disease [mh]
9. #4 OR #5 OR #6 OR #7 OR #8
10. Nigeria [mh] OR Nigeria* [tiab]
11. #1 AND #2 AND #9 AND #10
12. #1 AND #3 AND #9 AND #10
13. #2 AND #3 AND #9 AND #10
14. #1 AND #2 AND #3 AND #9 AND #10
15. #11 OR #12 OR #13 OR #14
16. Animals [mh] NOT Humans [mesh: noexp]
17. #15 NOT #16
18. "1970/01/01"[PDAT]: "2023/07/31"[PDAT]
19. #17 AND #18

**Google scholar search strategy**

“Noma”|Cancrum oris|stomatitis|“gangrenosa”|“NOMA”|“prevalence”|burden|“case fatality”|malnutrition|hepaciviru* HIV|poverty|“stomatitis gangrenosa”|“stomatitis gangrene*”|“ oro-facial gangrenous infection” children Nigeria.

**Cochrane central search strategy**

1. ("epidemiology"[Subheading] OR "epidemiology"[ tiab] OR "prevalence"[tiab] OR "prevalence"[MeSH Terms])
2. Noma [mh] OR Cancrum Oris [mh: noexp] OR Oris [mh] OR Cancrum [mh] OR stomatitis gangrenosa [tiab] OR NOMA [ tiab]
3. Malnutrition [mh] OR malnurished [mh] OR poverty [tiab] OR AIDS [tiab] OR “Acquired Immune Deficiency Syndrome” [tiab] OR “Acquired Immunodeficiency Syndrome” [tiab] OR Human Immunodeficiency Virus* [tiab] OR Malaria [tiab] OR measles [tiab] OR Chicken pox [tiab]
4. ("ulcerative gingivitis"[MeSH Terms] OR "noma therapy"[tiab] OR ("noma epidemiology"[ tiab]
5. "infections"[ tiab]) OR " oro-facial gangrenous infection "[ tiab])
6. children [mh] OR child [mh] OR under-five [mh] OR adolescent [mh] OR Chil* [tiab] OR infant* [tiab]
7. (neglected disease [tiab] OR noma etiology [tiab] OR oral hygienel [tiab]) AND (neglected diseas* [tiab] OR infection* [tiab])
8. Tropical disease [mh]
9. #4 OR #5 OR #6 OR #7 OR #8
10. Nigeria [mh] OR Nigeria* [tiab]
11. #1 AND #2 AND #9 AND #10
12. #1 AND #3 AND #9 AND #10
13. #2 AND #3 AND #9 AND #10
14. #1 AND #2 AND #3 AND #9 AND #10
15. #11 OR #12 OR #13 OR #14
16. Animals [mh] NOT Humans [mesh: noexp]
17. #15 NOT #16
18. "1970/01/01"[PDAT]: "2023/07/31"[PDAT]
19. #17 AND #18

**Supplemental File 2**

**Appendix 2: Risk of bias items and assessment of quality in the included studies**

| Questions | **Ratings** | **Points Scored** | Adeniyi | Bello | Denloye | Enwonwu | Farley | Fieger | Fomete | Idigbe | Oginni | Osuji | Otuyemi | Otuyemi |
| --- | --- | --- | --- | --- | --- | --- | --- | --- | --- | --- | --- | --- | --- | --- |
| **1. Was the study’s target population a close representation of the national population in relation to relevant variables?** | Yes (LOW RISK): The study’s target population was a close representation of the national population. | - | - | - | - | - | - | - | - | 0 |  |  |  |  |
|  | No (HIGH RISK): The study’s target population was clearly NOT representative of the national population. | 1 | 1 | 1 | 1 | 1 | 1 | 1 | 1 | - |  |  |  |  |
| **2. Was the sampling frame a true or close representation of the target population?** | Yes (LOW RISK): The sampling frame was a true or close representation of the target population. | 0 | - | - | - | - | - | - | - | 0 |  |  |  |  |
|  | No (HIGH RISK): The sampling frame was NOT a true or close representation of the target population. | 1 | - | 1 | - | - | 1 | - | - | - |  |  |  |  |
| **3. Was some form of random selection used to select the sample, OR, was a census undertaken?** | Yes (LOW RISK): A census was undertaken, OR, some form of random selection was used to select the sample (e.g. simple random sampling, stratified random sampling, cluster sampling, systematic sampling). | 0 | 0 |  | 0 | 0 | - | 0 | 0 | - |  |  |  |  |
|  | No (HIGH RISK): A census was NOT undertaken, AND some form of random selection was NOT used to select the sample. | 1 | - | 1 | - | - | 1 | - | - | 1 |  |  |  |  |
| **4. Was the likelihood of non-response bias minimal?** | Yes (LOW RISK): The response rate for the study was ≥75%, OR, an analysis was performed that showed no significant difference in relevant demographic characteristics between responders and non- responders | 0 | 0 | - | 0 | 0 | 0 | 0 | 0 | - |  |  |  |  |
|  | No (HIGH RISK): The response rate was <75%, and if any analysis comparing responders and non-responders was done, it showed a significant difference in relevant demographic characteristics between responders and non-responders | 1 | - | 1 | - | - | - | - | - | 1 |  |  |  |  |
| **5. Were data collected directly from the subjects (as opposed to a proxy)?** | Yes (LOW RISK): All data were collected directly from the subjects. | 0 | 0 | 0 | 0 | 0 | 0 | 0 | 0 | - |  |  |  |  |
|  | No (HIGH RISK): In some instances, data were collected from a proxy | 1 | - | - | - | - | - | - | - | 1 |  |  |  |  |
| **6. Was an acceptable case definition used in the study?** | Yes (LOW RISK): An acceptable case definition was used. | 0 | 0 | - | 0 | 0 | 0 | 0 | 0 | - |  |  |  |  |
|  | No (HIGH RISK): An acceptable case definition was NOT used | 1 |  | 1 | - | - | - | - | - | 1 |  |  |  |  |
| **7. Was the study instrument that measured the parameter of interest (e.g. prevalence of low back pain) shown to have reliability and validity (if necessary)?** | Yes (LOW RISK): The study instrument had been shown to have reliability and validity (if this was necessary), e.g. test-re- test, piloting, validation in a previous study, etc. | 0 | 0 |  | 0 | 0 | 0 | 0 | 0 | - |  |  |  |  |
|  | No (HIGH RISK): The study instrument had NOT been shown to have reliability or validity (if this was necessary). | 1 | - | 1 | - | - | - | - | - | 1 |  |  |  |  |
| **8. Was the same mode of data collection used for all subjects?** | Yes (LOW RISK): The same mode of data collection was used for all subjects. | 0 | 0 | 0 | 0 | 0 | 0 | 0 | 0 | - |  |  |  |  |
|  | No (HIGH RISK): The same mode of data collection was NOT used for all subjects. | 1 | - | - | - | - | - | - | - | 1 |  |  |  |  |
| **9. Were the numerator(s) and denominator(s) for the parameter of interest appropriate** | Yes (LOW RISK): The paper presented appropriate numerator(s) AND denominator(s) for the parameter of interest (e.g. the prevalence of low back pain). | 0 | 0 | - | 0 | 0 | 0 | 0 | 0 | - |  |  |  |  |
|  | No (HIGH RISK): The paper did present numerator(s) AND denominator(s) for the parameter of interest but one or more of these were inappropriate. | 1 | - | 1 | - | - | - | - | - | 1 |  |  |  |  |
| **10. Summary on the overall risk of study bias** | Low Risk (0 to 3)  Moderate Risk (4 to 6)  High Risk (7 to 9) | TOTAL | **4** | **7** | **1** | **1** | **2** | **4** | **4** | **7** |  |  |  |  |

**Supplemental File 3**

Appendix 3: **Characteristics of excluded studies**

| **Study ID** | **Reasons for Exclusion** |
| --- | --- |
| Adekeye et al  2012 [57] | No data about Noma prevalence or case fatality rate. |
| Adeola et al  2009 [58] | The study was a case series. No data about Noma prevalence or case fatality rate. |
| Aderinokun  1990 [59] | No data about Noma prevalence or case fatality rate. |
| Obiechina et al  2000 [60] | The study was a case series. No data about Noma prevalence or case fatality rate. |
| Oji  2002 [61] | The study was a case report. No data about Noma prevalence or case fatality rate. |
| Omeregie et al  2013 [62] | No data about Noma prevalence or case fatality rate. |
| Owobu et al  2022 [63] | The study was a case report. No data about Noma prevalence or case fatality rate. |
| Srour et al  2017 [64] | The study was a review article |
| Taiwo  1996 [65] | The study was a case report. No data about Noma prevalence or case fatality rate. |
